# Diminished activation of excitatory neurons in the prelimbic cortex leads to impaired working memory capacity in mice

**DOI:** 10.1186/s12915-023-01674-3

**Published:** 2023-08-11

**Authors:** Li-Xin Jiang, Geng-Di Huang, Yong-Lu Tian, Ri-Xu Cong, Xue Meng, Hua-Li Wang, Chen Zhang, Xin Yu

**Affiliations:** 1 Peking University Institute of Mental Health (Sixth Hospital), No.51 Huayuanbei Road, Haidian District, Beijing, 100191 China; 2 National Clinical Research Center for Mental Disorders & NHC Key Laboratory of Mental Health (Peking University), Beijing, 100191 China; 3 Beijing Municipal Key Laboratory for Translational Research On Diagnosis and Treatment of Dementia, Beijing, 100191 China; 4 Department of Addiction Medicine, Shenzhen Clinical Research Center for Mental Disorders, Shenzhen Mental Health Center, Shenzhen Kangning Hospital, No.77 Zhenbi Road, Pingshan District, Shenzhen, 518118 China; 5 Affiliated Mental Health Center, Southern University of Science and Technology, No.1088 Xueyuan Avenue, Fuguang Community, Taoyuan Street, Nanshan District, Shenzhen, 518118 China; 6 School of Psychological and Cognitive Sciences, Peking University, No.5 Summer Palace Road, Haidian District, Beijing, 100871 China; 7 IDG/McGovern Institute for Brain Research, Peking University, Beijing, 100871 China; 8 Key Laboratory of Cell Proliferation and Differentiation of the Ministry of Education, College of Life Sciences, Peking University, Beijing, 100871 China; 9 National Center of Gerontology, Beijing Hospital, No.1 Dahua Road, Dongdan, Dongcheng District, Beijing, 100005 China; 10 Beijing Key Laboratory of Neural Regeneration and Repair, Advanced Innovation Center for Human Brain Protection, School of Basic Medical Sciences, Capital Medical University, No.10 Xitoutiao, You’anmenwai, Fengtai District, Beijing, 100069 China

**Keywords:** Alzheimer's disease, Working memory capacity, 5XFAD mice, FOS, Excitatory neuron

## Abstract

**Background:**

Working memory capacity impairment is an early sign of Alzheimer's disease, but the underlying mechanisms remain unclear. Clarifying how working memory capacity is affected will help us better understand the pathological mechanism of Alzheimer's disease. We used the olfactory working memory capacity paradigm to evaluate memory capacity in 3-month-old 5XFAD (an animal model of Alzheimer's disease) mice. Immunofluorescence staining of the prefrontal cortex was performed to detect the number of FOS-positive neurons, calmodulin-dependent protein kinase II-positive neurons, and glutamate decarboxylase-positive neurons in the prelimbic cortex and infralimbic cortex. A chemogenetic method was then used to modulate the inhibition and activation of excitatory neurons in the prelimbic cortex of wild-type and 5XFAD mice and to measure the memory capacity of mice.

**Results:**

Working memory capacity was significantly diminished in 5XFAD mice compared to littermate wild-type mice. Neuronal activation of the prelimbic cortex, but not the infralimbic cortex, was attenuated in 5XFAD mice performing the olfactory working memory capacity task. Subsequently, the FOS-positive neurons were co-localized with both calmodulin-dependent protein kinase II-positive neurons and glutamate decarboxylase-positive neurons. The results showed that the activation of excitatory neurons in the prelimbic cortex was correlated with working memory capacity in mice. Our results further demonstrate that the chemogenetic inhibition of prelimbic cortex excitatory neurons resulted in reduced working memory capacity in wild-type mice, while the chemogenetic activation of prelimbic cortex excitatory neurons improved the working memory capacity of 5XFAD mice.

**Conclusion:**

The diminished activation of prelimbic cortex excitatory neurons in 5XFAD mice during task performance is associated with reduced working memory capacity, and activation modulation of excitatory neurons by chemogenetic methods can improve memory capacity impairment in 5XFAD mice. These findings may provide a new direction for exploring Alzheimer's disease therapeutic approaches.

**Supplementary Information:**

The online version contains supplementary material available at 10.1186/s12915-023-01674-3.

## Introduction

Alzheimer's disease (AD) is an age-dependent neurodegenerative brain disease that is the leading cause of dementia in the elderly population [1, 2]. The neuropathology of AD is characterized by extracellular Aβ plaques and intraneuronal neurofibrillary tangles accompanied by neuronal dysfunction/death and synapse loss [3–5]. The main clinical symptoms of AD are progressive memory loss and cognitive deterioration [6, 7]. As the disease progresses, AD patients gradually develop impairments in episodic memory, semantic memory, working memory (WM), and visuospatial memory [8–11]. Of these, impairment of WM is evident as early as in mild cognitive impairment, also referred to as the preclinical stage of AD, and worsens with progression to AD [12, 13]. Furthermore, WM deficits are central in normal neurocognitive aging and the rapid cognitive deterioration associated with dementias, such as AD [14, 15]. WM deficits in AD are thought to be responsible for several significant problems, such as difficulty dividing attention and manipulating remembered information [16, 17]. Compared to healthy controls, AD patients are more likely to be overloaded with information and exhibit rapid performance decline as task demands increase [18]. Therefore, it is of great interest to determine the mechanisms of WM impairment, which may provide a new direction for therapeutic approaches to AD.

WM is a vital process of the brain that actively maintains and processes information during a delay of a few seconds after sensory input is received and before behavior begins [19–22]. It is thought to be the core cognitive process that supports a range of behaviors, from perception to problem-solving and action control [23]. Working memory capacity (WMC) is a crucial component of WM and is the ability to retain information for a few seconds [19]. It is limited, and a restricted amount of information or number of items is actively retained in WM [24, 25]. Clarifying the mechanisms underlying the formation of WMC would facilitate the interpretation of the impairment of memory capacity in AD patients. Exploratory studies of WM mechanisms have focused more on the brain regions involved in WM and the electrical activity of neurons. The study of brain regions related to WM has shown that multiple regions of the brain are involved, and a great deal of research has shown that the prefrontal cortex (PFC) is critical for WM [26–28]. Disturbances to PFC activity during delays in the dorsolateral PFC in primates and medial PFC (mPFC) neurons in rodents impaired WM and WM-related activity [29–33]. Moreover, during behavior, neurons in the PFC exhibit highly diverse responses [34, 35].

In recent years, there has been increasing interest in how WM is affected in the early stages of AD. In our previous study, we developed a novel olfactory working memory capacity (OWMC) paradigm [36, 37] inspired by the rodent odor span task established by Dudchenko et al. [38] and Young et al. [39]. In this paradigm, we introduced a method for measuring the WMC of trial-specific information that we can use to assess the performance of mice from low to high loads of memory information. In this study, we will assess the impairment of WMC in 3-month-old 5XFAD (TG) mice using the OWMC paradigm. Previously, we found that 5XFAD mice have diminished neuronal activation in the mPFC while performing a task. We wanted to explore which subregions (prelimbic cortex [PrL] and infralimbic cortex [IL]) of the mPFC differ in neuronal activation during task performance. After specifying the brain regions associated with the OWMC task, we will go further to determine which type of neurons (excitatory and inhibitory) are involved in performing the task. Finally, we will apply a chemogenetic method to modulate the inhibition and activation of specific neurons in wild-type (WT) and 5XFAD mice to determine whether we can reduce WMC in WT mice and ameliorate WMC impairment in 5XFAD mice.

## Results

### WMC was significantly reduced in 3-month-old 5XFAD mice

**Fig. 1:**
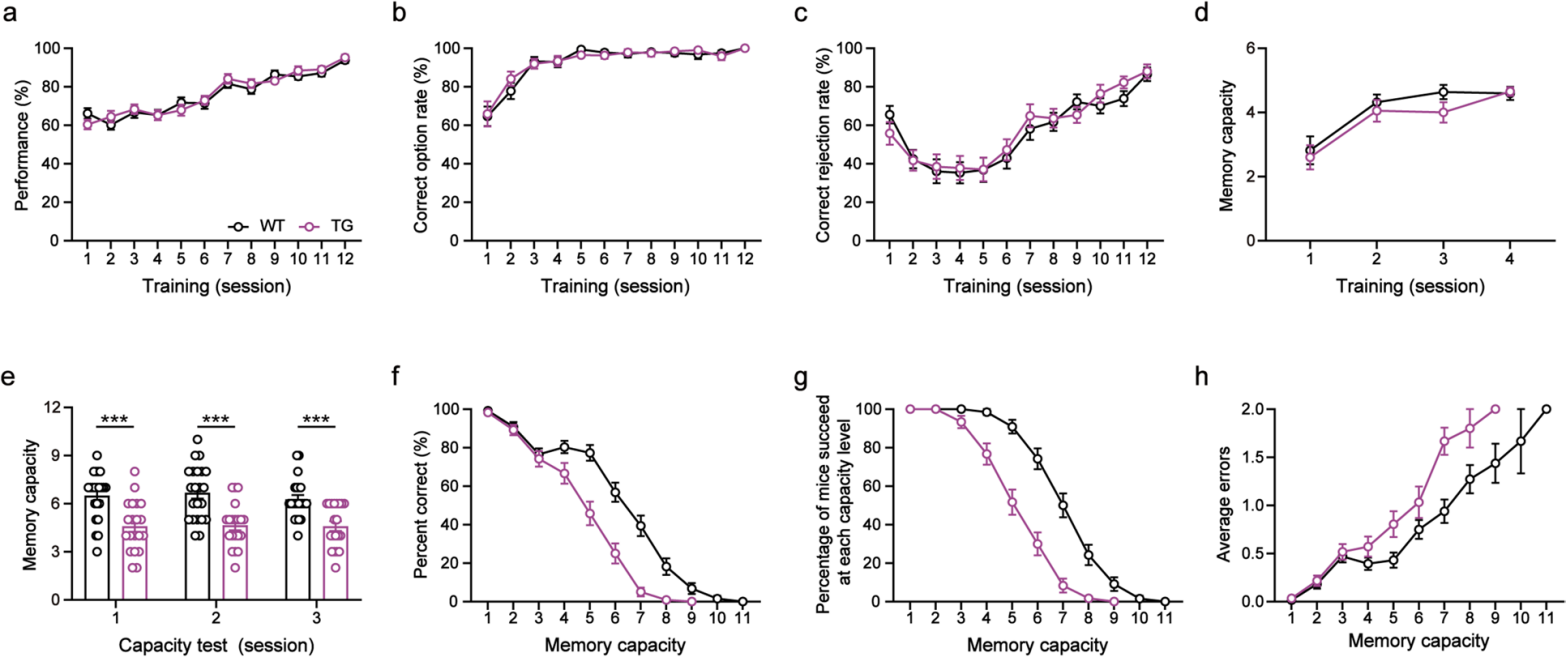
WMC was significantly reduced in 3-month-old 5XFAD mice compared to littermate WT mice. **a-c** Performance correct rate, correct option rate and correct rejection rate of mice during the NMSS-rule learning phase (Two-way ANOVA, all *p* > 0.05). **d** WMC of mice during the NMMS-rule learning phase (Two-way ANOVA, *p* > 0.05).** e** WMC of mice during the test phase (Unpaired Student’s *t* test, ***p < 0.001). **f** Percent correct at each capacity level (Two-way ANOVA, ****p < 0.0001). **g** Percentage of mice succeeded at each capacity level (Two-way ANOVA, ****p < 0.0001). **h** Average errors at each capacity level (Two-way ANOVA, ****p < 0.0001). WT: *n* = 22; TG: *n* = 20. Data are presented as the mean ± SEM

At the beginning of this study, we used the OWMC paradigm to assess the WMC of 3-month-old 5XFAD mice. During the nonmatching to a single odor sample (NMSS) rule-learning phase, we recorded the responses of mice for each trial and counted the performance accuracy (Fig. 1a), correct selection rate (Fig. 1b), and correct rejection rate (Fig. 1c). There was no difference between WT and 5XFAD mice in these indices (all *p* > 0.05). Next, for 4 days of nonmatching to multiple odor samples (NMMS) rule-learning, we measured the daily memory capacity of mice and found that WT and 5XFAD mice showed similar WMC (*p* > 0.05, Fig. 1d). It was clear that both 5XFAD and WT mice could learn the NMSS and NMMS rules well. Finally, the capacity test was performed for 3 days. We recorded the WMC of each mouse (Fig. 1e). We counted the accuracy of mice with different memory capacities (Fig. 1f) as well as the average number of errors (Fig. 1h), and we calculated the percentage of mice that could complete different memory capacity tasks (Fig. 1g). We found that the WMC was significantly diminished in 5XFAD mice (Day-1: *t* = 3.96, *p* < 0.001; Day-2: *t* = 4.22, *p* < 0.001; Day-3: *t* = 4.18, *p* < 0.001) compared to littermate WT mice. As the memory load increased, 5XFAD mice made significantly more errors than WT mice (*F*_(1, 819)_ = 18.13, *p* < 0.0001) and had a significantly lower accuracy (*F*_(1, 1254)_ = 84.52, *p* < 0.0001). Additionally, 5XFAD mice completed the task at a significantly lower percentage than WT mice (*F*_(1, 1254)_ = 137.10, *p* < 0.0001). Therefore, we conclude that the WMC of 3-month-old 5XFAD mice is significantly lower than that of WT mice.

### Neuronal activation of the PrL was attenuated in 5XFAD mice performing the OWMC task

**Fig. 2:**
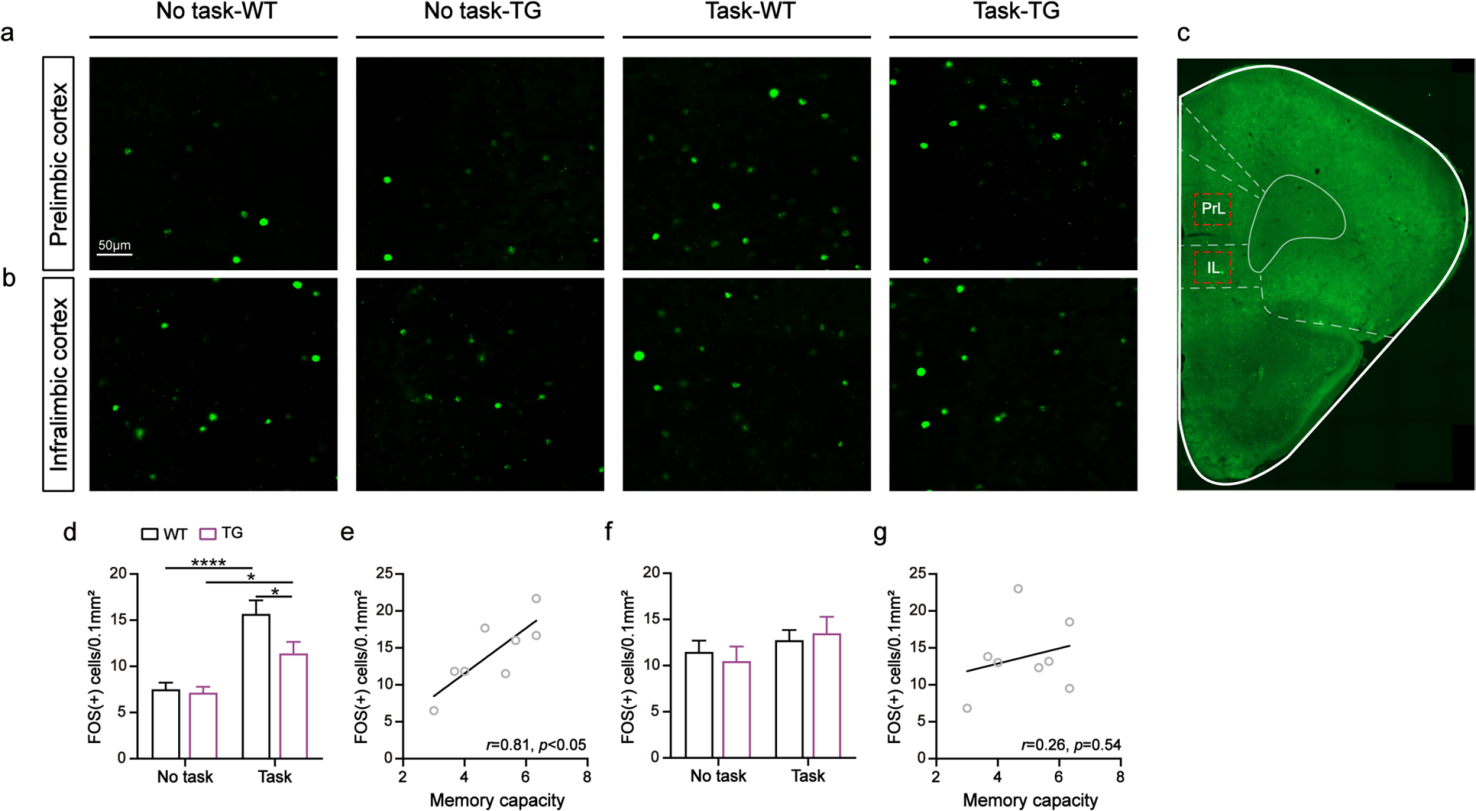
The activation of PrL neurons was associated with WMC. **a,b** Representative images show the FOS^+^ neurons in the PrL and IL of each group of mice. **c** Representative image show the PrL and IL. **d** The number of FOS^+^ neurons in the PrL (Two-way ANOVA, interaction: *p* > 0.05, task:* p* < 0.0001, genotype:* p* < 0.05; post hoc Tukey’s tests, *p < 0.05, ****p < 0.0001). *n* = 12 slices from four mice per group. **e** Relationship between the number of FOS^+^ neurons in the PrL and WMC in the task group mice (Linear regression, *p < 0.05). *n* = 8. **f** The number of FOS^+^ neurons in the IL (Two-way ANOVA, all *p* > 0.05; post hoc Tukey’s tests, all* p* > 0.05). *n* = 12 slices from four mice per group. **g** Relationship between the number of FOS^+^ neurons in the IL and WMC of in the task group mice (Linear regression, *p* > 0.05). *n* = 8. Data are presented as the mean ± SEM

In a previous study [36], we found that neuronal activation of the mPFC was attenuated in 5XFAD mice while performing a task. We wanted to explore which subregions of the mPFC (the PrL, IL, or both; Fig. 2c) have activation differences in neurons while performing the task. The mice were divided into 4 groups: WT + no OWMC task, 5XFAD + no OWMC task, WT + OWMC task, and 5XFAD + OWMC task. We calculated the number of FOS-positive (FOS^+^) neurons in the two brain regions (Fig. 2a, b). We then found that the number of FOS^+^ neurons in the PrL were significantly higher in the task group mice than in the nontask group mice (task: *F*_(1, 44)_ = 32.19, *p* < 0.0001; Fig. 2d), but not in the IL (task: *F*_(1, 44)_ = 2.19, *p* > 0.05; Fig. 2f). In the task group, the number of FOS^+^ neurons in the PrL were significantly lower in 5XFAD mice than in WT mice (genotype: *F*_(1, 44)_ = 4.50, *p* < 0.05; Task-TG vs. Task-WT: *p* < 0.05, Tukey’s tests; Fig. 2d). We also conducted a correlation analysis between the number of FOS^+^ neurons and the WMC of task group mice. We found a positive correlation between the number of FOS^+^ neurons in the PrL and WMC (*r* = 0.81, *p* < 0.05; Fig. 2e), while there was no correlation between the number of FOS + neurons in the IL and WMC (*r* = 0.26, *p* > 0.05; Fig. 2g). These results indicate that 5XFAD mice exhibit diminished neuronal activation of the PrL when performing the OWMC task.

### Activation of excitatory neurons was associated with WMC

**Fig. 3:**
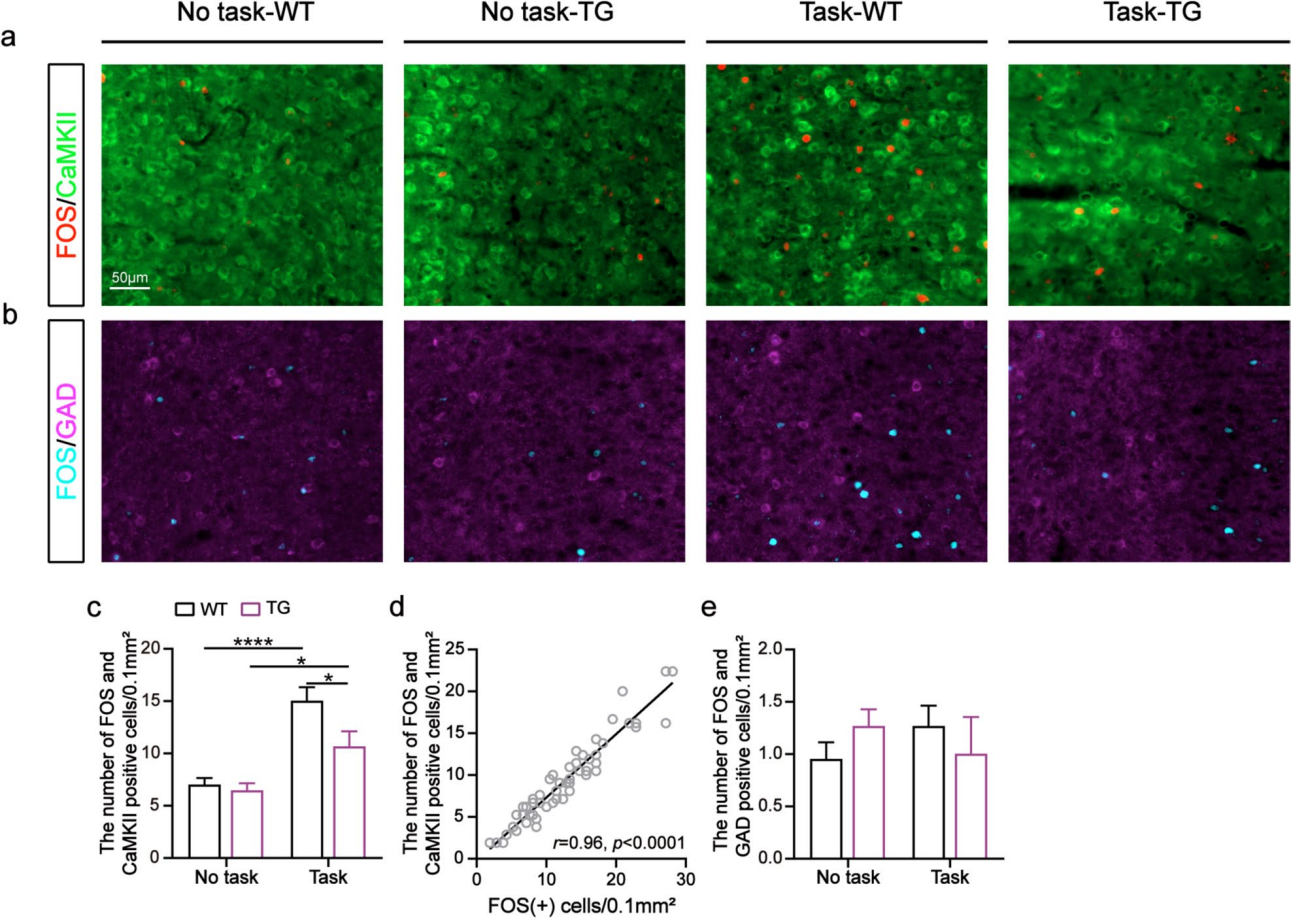
The activation of excitatory neurons was associated with WMC. **a** Representative images showing the FOS^+^ neurons, CaMKII^+^ neurons and co-localized cells in the PrL of each group of mice. **b** Representative images showing the FOS^+^ neurons, GAD^+^ neurons and co-localized cells in the PrL of each group of mice. **c** The number of FOS^+^ neurons co-localized with CaMKII^+^ neurons (Two-way ANOVA, interaction: *p* > 0.05, task:* p* < 0.0001, genotype:* p* < 0.05; post hoc Tukey’s tests, *p < 0.05, ****p < 0.0001). No task: *n* = 15 slices from five mice per group; Task: *n* = 12 slices from four mice per group. **d** Relationship between the amount of FOS^+^ neurons and FOS^+^ neurons co-localized with CaMKII^+^ neurons (Linear regression, ****p < 0.0001). *n* = 54 slices from eighteen mice. **e** The number of FOS^+^ neurons co-localized with GAD^+^ neurons (Two-way ANOVA, all *p* > 0.05; post hoc Tukey’s tests, all* p* > 0.05). *n* = 9 slices from three mice per group. Data are presented as the mean ± SEM

Different types of neurons in the mPFC play different roles in encoding and regulating behaviors [40]. To investigate which specific types of neurons are associated with WMC, we performed double immunostaining against FOS and the marker of excitatory neurons (CaMKII, Fig. 3a) or inhibitory neurons (GAD, Fig. 3b). We found that the number of FOS^+^ neurons co-localized with calmodulin-dependent protein kinase II-positive (CaMKII^+^) neurons was significantly higher in the task group of WT mice than in the task group of 5XFAD mice (genotype: *F*_(1, 50)_ = 5.88, *p* < 0.05; Task-TG vs. Task-WT: *p* < 0.05, Tukey’s tests; Fig. 3c). Furthermore, a linear relationship was observed between the number of FOS^+^ neurons and the number of FOS^+^ neurons co-localized with CaMKII^+^ neurons (*r* = 0.96, *p* < 0.0001; Fig. 3d). As the number of FOS^+^ neurons increased, there was a concomitant increase in the number of FOS^+^ neurons that co-localized with CaMKII^+^ neurons. However, we did not detect a difference in the number of FOS^+^ neurons co-localized with glutamate decarboxylase-positive (GAD^+^) neurons between the four groups (task: *F*_(1, 32)_ = 0.01, *p* > 0.05; genotype: *F*_(1, 32)_ = 0.01, *p* > 0.05; Fig. 3e). Thus, we suggest that WMC is associated with the activation of excitatory neurons in the PrL.

### Chemogenetic inhibition of PrL excitatory neurons attenuated the WMC of WT mice

**Fig. 4:**
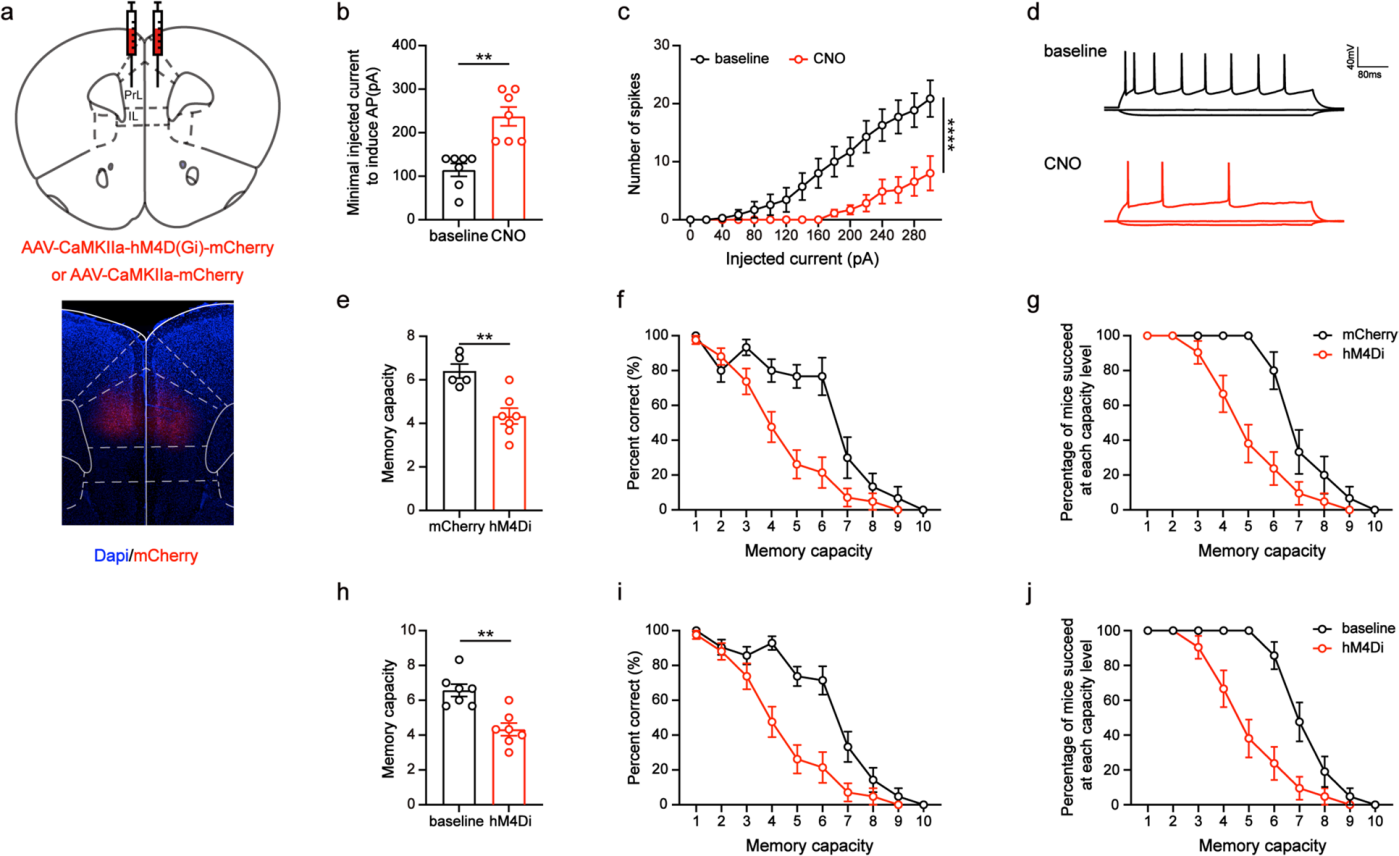
Chemogenetic inhibition of PrL excitatory neurons attenuated the WMC of WT mice. **a** Schematic diagram (coronal section) showing the injection target of the virus (hM4Di-mCherry or mCherry) in the PrL. **B** The minimal injected current to induce action potential (Paired Student’s *t* test, **p < 0.01). *n* = 7. **c** The number of induced action potentials at different current steps (Two-way ANOVA, ****p < 0.0001). **d** Current–voltage relationship of a representative PrL neuron recorded before and during CNO perfusion. **e** The WMC of mice in the mCherry and hM4Di group (Unpaired Student’s *t* test, **p < 0.01). mCherry: *n* = 5; hM4Di: *n* = 7. **f** Percent correct at each capacity level of mice in the mCherry and hM4Di group (Two-way ANOVA, ****p < 0.0001). **g** Percentage of mice succeeded at each capacity level in the mCherry and hM4Di group (Two-way ANOVA, ****p < 0.0001). **h** The WMC of mice in the test phase before and during CNO perfusion (Paired Student’s *t* test, **p < 0.01). *n* = 7. **i** Percent correct at each capacity level of mice before and during CNO perfusion (Two-way ANOVA, ****p < 0.0001). **j** Percentage of mice succeeded at each capacity level before and during CNO perfusion (Two-way ANOVA, ****p < 0.0001). Data are presented as the mean ± SEM

**Fig. 5:**
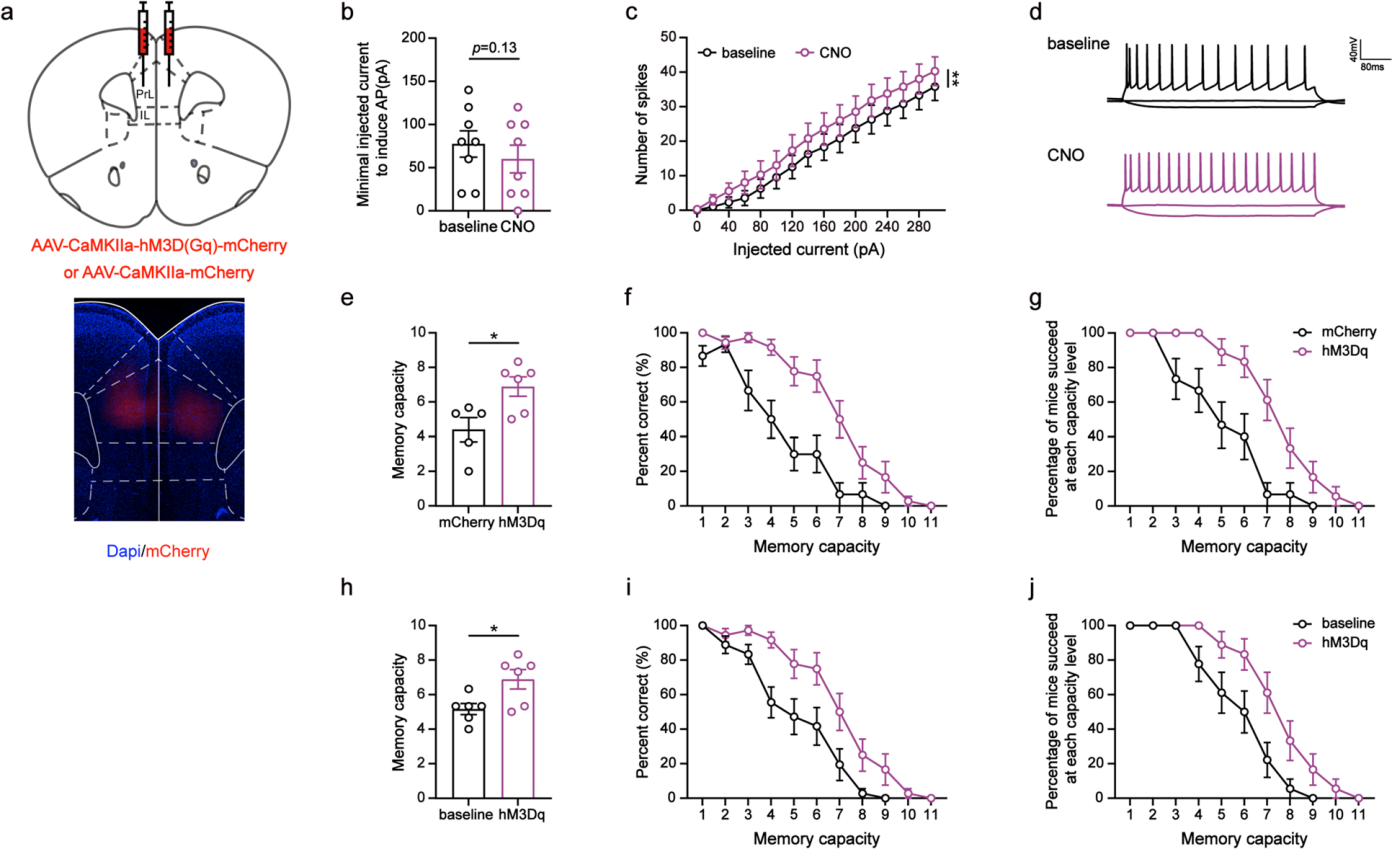
Chemogenetic activation of PrL excitatory neurons improved the WMC of 5XFAD mice. **a** Schematic diagram (coronal section) showing the injection target of the virus (hM3Dq-mCherry or mCherry) in the PrL. **B** The minimal injected current to induce action potential (Paired Student’s *t* test, *p* = 0.13). *n* = 8. **c** The number of induced action potentials at different current steps (Two-way ANOVA, **p < 0.01). **d** Current–voltage relationship of a representative PrL neuron recorded before and during CNO perfusion. **e** The WMC of mice in the mCherry and hM3Dq group (Unpaired Student’s *t* test, *p < 0.05). mCherry: *n* = 5; hM3Dq: *n* = 6. **f** Percent correct at each capacity level of mice in the mCherry and hM3Dq group (Two-way ANOVA, ****p < 0.0001). **g** Percentage of mice succeeded at each capacity level in the mCherry and hM3Dq group (Two-way ANOVA, ****p < 0.0001). **h** The WMC of mice in the test phase before and during CNO perfusion (Paired Student’s *t* test, *p < 0.05). *n* = 6. **i** Percent correct at each capacity level of mice before and during CNO perfusion (Two-way ANOVA, ****p < 0.0001). **j** Percentage of mice succeeded at each capacity level before and during CNO perfusion (Two-way ANOVA, ****p < 0.0001). Data are presented as the mean ± SEM

To further test whether PrL excitatory neurons are associated with WMC, the DREADD (designer receptors exclusively activated by designer drugs)-based tools were used. We bilaterally injected the inhibitory virus (hM4Di-mCherry) or non-functional control virus (mCherry) into the PrL of WT mice (Fig. 4a). To ensure that the virus (hM4Di-mCherry) was effective, whole-cell recordings were performed in PrL neurons (Fig. 4d). The administration of Clozapine-N-oxide (CNO) inhibited the activity of PrL neurons expressing hM4Di, which led to an increase in the minimum required injection current for the induction of action potentials (*t* = 4.37, *p* < 0.01; Fig. 4b) and a reduction in the number of spikes (*F*_(1, 207)_ = 82.60, *p* < 0.0001; Fig. 4c). To establish a baseline before administering CNO (saline injection), we conducted a three-day capacity test on mice injected with hM4Di. Subsequently, we administered CNO to both hM4Di-injected and mCherry-injected mice and evaluated their WMC. Compared to mice injected with mCherry, we observed a significant decrease in WMC in mice injected with hM4Di (*t* = 4.04, *p* < 0.01; Fig. 4e). As the memory load increased, the percent correct in the hM4Di injection group was significantly lower than that of the control group (injected with mCherry, *F*_(1, 328)_ = 39.89, *p* < 0.0001; Fig. 4f), and the percentage of task completion was also lower (*F*_(1, 328)_ = 44.19, *p* < 0.0001; Fig. 4g). Similarly, we found that the WT mice with inhibited excitatory neuronal activity in the PrL showed a significant decrease in memory capacity (*t* = 3.92, *p* < 0.01; Fig. 5h) compared to their baseline. As the memory load increased, the percent correct decreased significantly after excitatory neuronal activity was inhibited (*F*_(1, 388)_ = 57.99, *p* < 0.0001; Fig. 4i). Additionally, they completed the task at a significantly lower percentage than before (*F*_(1, 388)_ = 64.37, *p* < 0.0001; Fig. 4j). These results suggest that inhibiting the activity of PrL excitatory neurons can attenuate the WMC in WT mice.

### Chemogenetic activation of PrL excitatory neurons improved the WMC of 5XFAD mice

We hypothesized that if the inhibition of PrL excitatory neuronal activity is responsible for reduced WMC, then activating these neurons may ameliorate WMC impairment in 5XFAD mice. To test our hypothesis, we bilaterally injected excitatory virus (hM3Dq-mCherry) or mCherry encoding virus into the PrL of 5XFAD mice (Fig. 5a). As with WT mice, the effectiveness of the virus (hM3Dq-mCherry) was also verified by electrophysiological recordings (Fig. 5d). CNO administration activated the activity of hM3Dq-expressing PrL neurons, resulting in an increase in the number of spikes (*F*_(1, 239)_ = 10.80, *p* < 0.01; Fig. 5c) and a relative decrease in the minimum injection current required to induce action potentials (*t* = 1.70, *p* = 0.13; Fig. 5b). Initially, we examined the WMC of 5XFAD mice under natural conditions (saline injection) for three days. Next, we injected CNO into the 5XFAD mice with hM3Dq injection or mCherry injection and tested their WMC. Compared to the mCherry injection group, the hM3Dq injection group of 5XFAD mice exhibited a higher WMC (*t* = 2.82, *p* < 0.05; Fig. 5e). Additionally, as the memory load increased, the 5XFAD mice injected with hM3Dq showed higher rates of accuracy (*F*_(1, 321)_ = 65.91, *p* < 0.0001; Fig. 5f) and task completion (*F*_(1, 321)_ = 51.11, *p* < 0.0001; Fig. 5g). Furthermore, our findings showed that activating excitatory neuronal activity in the PrL led to significantly improved memory capacity in 5XFAD mice compared to baseline (*t* = 3.97, *p* < 0.05; Fig. 5h). As the memory load increased, their percent correct increased significantly (*F*_(1, 348)_ = 41.42, *p* < 0.0001; Fig. 5i) after excitatory neuronal activity was activated, and they completed the task at a higher percentage than before (*F*_(1, 348)_ = 28.76, *p* < 0.0001; Fig. 5j). Therefore, our results suggest that activating excitatory neurons in the PrL can ameliorate the WMC deficits in 5XFAD mice.

## Discussion

In recent years, the neurobiological mechanisms behind WM have received increasing attention. For different disorders, such as schizophrenia, AD, and attention deficit and hyperactivity disorder, the mechanisms of WM impairment may differ. Memory capacity is an essential component of WM. However, the neurobiological mechanisms behind WMC have yet to be well explored due to the lack of appropriate and effective behavioral paradigms for animals. Our previous work established a more sensitive and robust paradigm (the OWMC paradigm) for measuring WMC. In the present study, we used this paradigm to assess memory capacity impairment in 3-month-old 5XFAD mice and found that the WMC of 5XFAD mice was significantly lower than that of WT mice. By exploring the mechanisms responsible for this impairment, we found that the impairment of memory capacity in 5XFAD mice was associated with diminished activation of excitatory neurons in the PrL. To further demonstrate that attenuated activation of excitatory neurons in the PrL is associated with reduced WMC, we intervened by a chemogenetic method. We found that inhibition of excitatory neurons in the PrL impaired WMC in WT mice, whereas activation of excitatory neurons in the PrL ameliorated WMC impairment in 5XFAD mice.

5XFAD mice, which are widely used, recapitulate many AD-related phenotypes and have a relatively early and aggressive presentation. The mice accumulate high levels of intraneuronal Aβ_42_ at approximately 1.5 months of age, and extracellular amyloid deposition begins at approximately 2 months [41]. 5XFAD mice show neuropathological changes very early, while cognitive impairment appears much later. The impairment of recognition memory and spatial reference memory in 5XFAD mice generally appears at 4–5 months of age [41–44]. In this study, we detected a significant decrease in the WMC of 3-month-old 5XFAD mice using the OWMC task. Thus, we demonstrated that the OWMC paradigm has higher sensitivity than the common behavioral paradigm (i.e., novel object recognition test, Y-maze, and Morris water maze) for assessing cognitive function. This paradigm is more complex than the common behavioral paradigm. We trained mice over a long period to better learn the rules of the task. In the OWMC task, the sample odor for each trial is independent of the previous trial. The mice need to maintain increasing information from the list of odors and also use this information flexibly to make appropriate responses. During the testing phase, this paradigm can continuously increase the task's difficulty until the experimental animal fails to complete it. Thus, this paradigm can be more sensitive to detect changes in cognitive function.

We detected impairment of WMC in 5XFAD mice at an earlier age (3 months) through the OWMC paradigm. This led us to explore the neurobiological mechanisms involved. We first focused on the brain regions involved in WMC. In previous studies, several brain regions have been reported to be associated with the storage of WM information (i.e., the PFC [19, 45–47], sensory cortex [48, 49], and posterior parietal cortex [50, 51]), with the most extensive studies on the PFC. In our previous study [36], we also found that 5XFAD mice had diminished neuronal activation in the mPFC when performing OWMC tasks. In the present study, we wanted to further clarify the activation of neurons in two subregions (the PrL and IL) of the mPFC when performing this task paradigm. Neuronal activation was attenuated in the PrL of 5XFAD mice but not in the IL. WM, as a cognitive process, requires the integration of transient sensory inputs over time and the maintenance of task-relevant stimulus representations [52]. Differences in attention, motivation, and motion can serve as confounding factors in WM assessments. If 5XFAD mice exhibit lower motivation, reduced reward responsiveness, and decreased activity, it could potentially lead to poorer task performance. Prior to training, we recorded the body weight and daily food intake of the mice and found no differences between the 5XFAD and WT groups in these aspects [36]. Additionally, we implemented strict food restriction and provided a minimal amount of reward food pellets (0.05 g) to ensure the mice's motivation. At the end of each day's session, we provided food to the mice and observed that they consumed it quickly, indicating a strong motivation to eat even after the experiment. From the experimental results, it can be seen that there were no differences in performance between the 5XFAD and WT mice during the NMSS and NMMS training stages, further ruling out the possibility of differences in motivation, arousal, or reward processing between the two groups. Therefore, we propose that the attenuated activation of PrL neurons leads to the reduced WMC in the 5XFAD mice, rather than affecting their WMC through motivation, arousal, or reward processing. Previous evidence suggests that the PrL and IL regions can be functionally separated. In line with its connection to affective and autonomic structures, the IL appears to play a specific role in regulating fear-related behaviors, especially those related to eliminating emotional responses [53–55]. Compared to the functions of the IL, the PrL region is thought to be less involved in emotional aspects but more involved in cognitive functions, such as WM [35, 56–60]. Our findings are consistent with previous studies and provide further evidence of the importance of the PrL for WMC.

Different types of neurons in the mPFC play different roles in encoding and regulating behavior [61, 62]. The mPFC in rodents is primarily composed of a majority of excitatory glutamatergic pyramidal neurons and 15%-20% of inhibitory GABAergic interneurons. The function of the PFC relies on a delicate balance between excitation and inhibition, mediated by excitatory pyramidal neurons and various GABAergic interneurons [63, 64]. The majority (80%) of cortical interneurons are vasoactive intestinal polypeptide, parvalbumin, and somatostatin neurons [65]. These different types of interneurons are differentially regulated by sensory stimuli, motor behaviors, brain states, and neuromodulatory inputs [66, 67]. Therefore, different types of interneurons may play distinct roles in local computation and exert unique control over excitatory output [65, 68]. When assessing the cause to the cellular level, we found that the activation of PrL excitatory neurons was attenuated in 5XFAD mice performing the OWMC task. The immunohistochemistry results did not reveal any changes in inhibitory interneurons. We believe that this may be due to the relatively small proportion of inhibitory interneurons and their differentiation into multiple types, each with its own functions. As a result, immunohistochemistry may not effectively capture the activation of certain types or subsets of inhibitory interneurons.

Extracellular amyloid deposition in 5XFAD mice begins at approximately 2 months, first in layer V of the cortex and subiculum, and increases rapidly with age [41]. By 3 months of age, significant amyloid deposition was already present in the mPFC [36]. Soluble Aβ oligomers and amyloid plaques can alter the function of local neuronal circuits and large-scale networks by disrupting the balance of synaptic excitation and inhibition (E/I balance) in the brain [69]. Previous studies have found that the functional state of the PrL depends mainly on the coordinated activity of glutamatergic pyramidal neurons. This activity heavily depends on the cellular E/I dynamic balance [70, 71]. Furthermore, the imbalance of pyramidal neuron E/I is thought to contribute to many of the symptoms observed in neuropsychiatric disorders [71–73]. As early as 1995, Goldman-Rakic et al. proposed that WM depends on the ability of pyramidal neuron networks to fire continuously [74]. In addition, Tian et al. found that excitatory neurons in the PrL exhibited emergent properties in a context-dependent manner underlying a short-term memory-like behavior paradigm, which was not observed in 5XFAD mice [75]. From this, we can infer that the diminished activation of PrL excitatory neurons in 5XFAD mice during task performance is associated with reduced WMC. To strengthen this hypothesis, we used a chemogenetic method to modulate the inhibition and activation of excitatory neurons in WT and 5XFAD mice. As expected, inhibition of PrL excitatory neurons in WT mice reduced their WMC, and activation of PrL excitatory neurons in 5XFAD mice improved the impairment of their WMC. Therefore, we demonstrate that attenuated activation of PrL excitatory neurons in 5XFAD mice leads to a decrease in their WMC.

This study also has some limitations. We used 5XFAD mice to explore the neurobiological mechanisms of WM impairment in AD. We found that PrL excitatory neuron activation was diminished in 5XFAD mice. Is this phenomenon only present in 5XFAD mice, or is it also true in other AD models? We have yet to answer this question, which requires further studies. Previous behavioral research studies have predominantly been conducted on male mice. In order to establish a better comparative reference with these studies, only male mice were selected for our research. Therefore, we cannot be certain whether gender differences will have an impact on the results of our study. Moreover, in the present study, we did not detect neuronal electrical activity in the PrL brain region while the mice were performing the task, so we do not know the difference in neuronal firing activity between the two types of mice.

## Conclusions

We found that diminished activation of excitatory neurons in the PrL led to impaired WMC in mice. These findings further extend to understanding the neurobiological mechanism of WM impairment in AD. Moreover, a possible intervention direction can be proposed: can WM impairment in AD be ameliorated by activating excitatory neuron firing? We need to explore this further.

## Methods

### Animals

5XFAD mice (overexpressing K670N/M671L + I716V + V717I mutations of human APP and M146L + L286V mutations of human PS1) were purchased from Jackson Laboratory (Bar Harbor, ME, USA, strain no. 008730). The WT littermates were used as controls. Age (3 months) and body weight were kept constant among experimental control factors. Mice were housed in a temperature- and humidity-controlled environment (22 ± 2 °C, 40–70%) with a 12-h light/12-h dark cycle. Prior to experiments, food and water were freely available. In this study, only male mice were used. All animal experimental procedures were conducted in accordance with the Guide for the Care and Use of Laboratory Animals (8th edition) and were approved by the Institutional Animal Care and Use Committee of Peking University.

### OWMC task

**Fig. 6:**
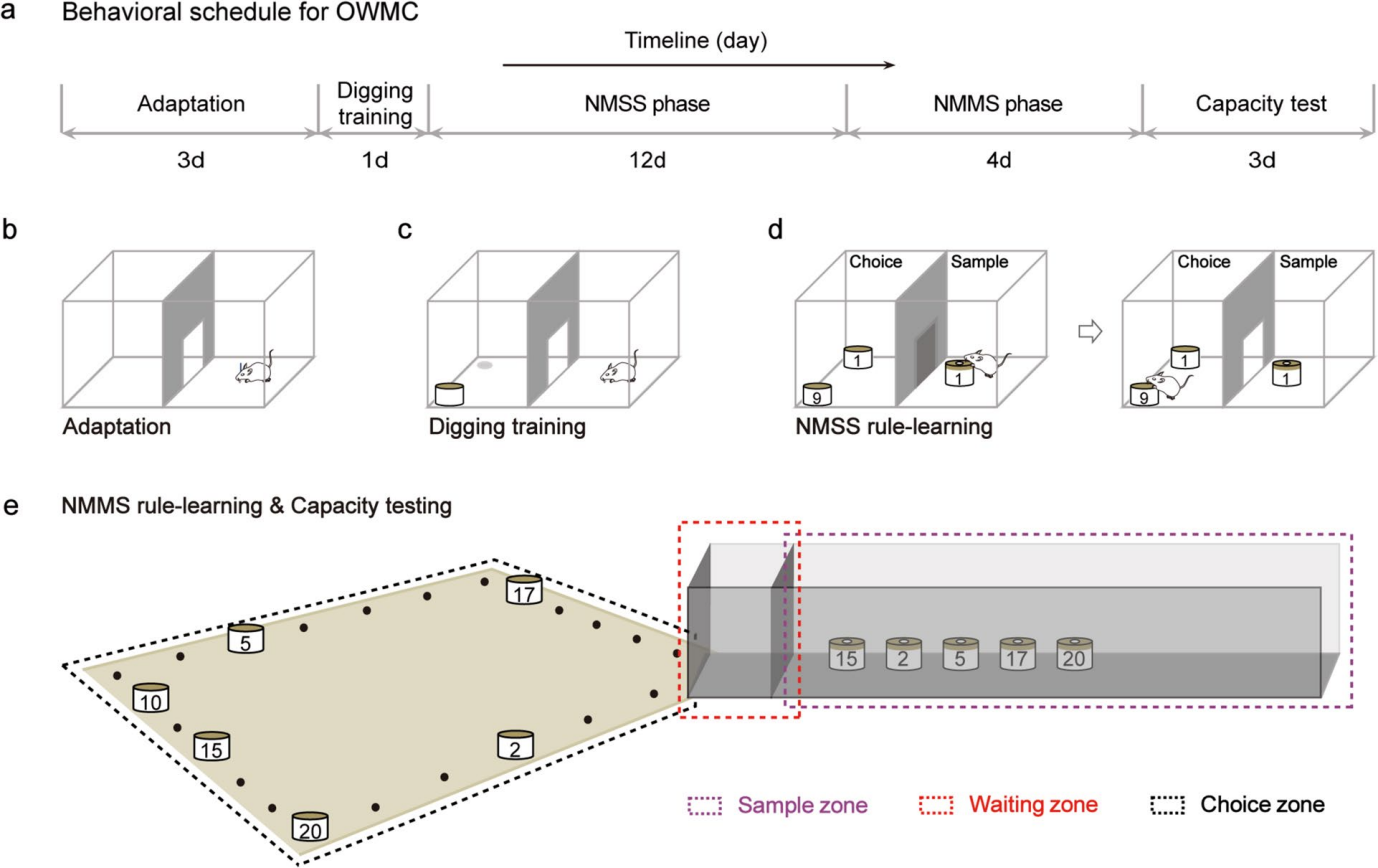
Schematic diagram of the OWMC task. **a** Timeline of the OWMC task. **b** Context adaptation of the OWMC task. **c** Digging training phase of the OWMC task. **d** NMSS rule-learning phase of the OWMC task. **e** NMMS rule-learning and capacity testing phase of the OWMC task

The OWMC task involves five stages: context adaptation, digging training, NMSS rule-learning, NMMS rule-learning, and capacity testing (Fig. 6a). In the first stage, mice are handled to reduce stress and acclimate to the training cage (Fig. 6b). The second stage involves training mice to dig in a bowl of unscented sawdust to locate a piece of cheese (Fig. 6c). In the third stage, mice are trained to find cheese pellets in a bowl with a novel odor (Fig. 6d). The fourth stage requires mice to discern the novel odor among multiple scented bowls to find the cheese pellet (Fig. 6e). Finally, in the fifth stage, mice undergo several WMC tests until they achieve a stable level of performance (Fig. 6e). For further detail regarding this paradigm, please refer to our previously published article [37].

### Tissue preparation

After 24 h of the capacity test, 5XFAD mice and littermates were randomly divided into 4 groups (5XFAD + OWMC task, WT + OWMC task, 5XFAD + non-OWMC task, WT + non-OWMC task). Mice in the task groups performed 2 capacity test trials, a 3-sample and a 6-sample trial, while mice in the nontask groups entered a cage and freely detected the same odors as the task groups. Then, the mice in the nontask group entered the empty sample zone and received a piece of cheese in the empty selection zone. Ninety minutes after the behavioral assessments, mice were deeply anesthetized and perfused with 30 ml of 0.9% saline via the cardiac artery, followed by 30 ml of 4% paraformaldehyde (PFA). The brains were removed and immersed in 10 ml of 4% PFA at 4 °C for 24 h. The PFC region of the brains was sliced into 30-μm-thick sections from the coronal plane with a freezing microtome. Sections were collected for immunohistochemistry.

### Immunohistochemistry and microscopy

Slices were washed 3 times with 0.1 M PBS for 5 min to remove the OCT. The slices were subsequently permeabilized and blocked in 0.1 M PBS containing 0.3% Triton X-100 and 5% bovine serum albumin (BSA) for 1 h at room temperature (RT). Next, slices were incubated overnight with the primary antibodies (anti-c-FOS, 1:1000, 2250 s, Cell Signaling Technology; anti-c-FOS, 1:1000, ab208942, Abcam; anti-CaMKII, 1:1000, ab52476, Abcam; anti-GAD65 + GAD67, 1:1000, ab183999, Abcam) diluted in blocking solution at 4 °C, and unbound antibody was removed by rinsing with 0.1 M PBS. Then, the brain slices were incubated in species-specific secondary antibodies (Alexa Fluor 488-conjugated goat anti-rabbit, 1:500, ab150077, Abcam; Alexa Fluor 594-conjugated goat anti-mouse, 1:500, ab150116, Abcam) diluted in 0.1 M PBS with 5% BSA for 1 h at RT. Finally, the slices were washed 3 times with 0.1 M PBS for 5 min each to remove excess secondary antibodies, and the nuclei were stained with DAPI (1:1000; Sigma Chemical) for 20 min at RT. Images were acquired using the Zeiss Axio Scan.Z1 Digital Slide Scanner. In this study, we utilized an aplan apochromat 20 × /0.8 M27 objective for imaging. The light source employed was HXP120V. The beam filters utilized were as follows: Filter 395 (Excitation: 330–375 nm; Emission: 430–470 nm), Filter 498 (Excitation: 453–485 nm; Emission: 507–546 nm), and Filter 603 (Excitation: 581–593 nm; Emission: 618–675 nm). The imaging device used was the Orca Flash 4.0. The acquired images have a bit depth of 16 bits, and we utilized Zen Blue 2 software to capture images of specific brain regions. Three slices from each mouse were selected for analysis. Furthermore, ImageJ was used to count and analyze the number of immunopositive cells. The scale bars are 50 μm, and each image area is 350 μm × 300 μm.

### Virus injection

The mice were anesthetized with tribromoethanol (240 mg/kg, Sigma) and then fixed on a stereotaxic frame (RWD Life Science, Shenzhen, China). After horizontal adjustment of the skull position under a stereomicroscope (RWD Life Science, Shenzhen, China), a minor craniotomy was performed with a thin drill over the PrL (typical coordinate: 1.98 mm anterior to Bregma; 0.32 mm lateral to the midline). Adeno-associated virus (AAV) carrying the hM3D (AAV-CaMKIIα-hM3D(Gq)–mCherry) and hM4D (AAV-CaMKIIα-hM4D(Gi)-mCherry) fusion genes or mcherry (AAV-CaMKIIα -mcherry) was injected through a microsyringe pump at 40 nL/min using a 5 μL microsyringe (#700, Hamilton, USA). A total of 80 nL of the virus was injected at a depth of 2.05 mm from the Bregma. At the end of the infusion, the pipette was held for 10 min to allow the virus to spread. hM3Dq (TG: *n* = 6) was injected for activation, and hM4Di (WT: *n* = 7) was injected for inhibition by bilateral injection. Control experiments were conducted using adenovirus expressing only mCherry and bilaterally injected in both WT (*n* = 5) and TG (*n* = 5) subjects. The viruses hM3Dq (AAV_2/9_, titer: 2.0 × 10^12^ v.g./mL) and hM4Di (AAV_2/9_, titer: 2.0 × 10^12^ v.g./mL) or mCherry (AAV_2/9_, titer: 2.0 × 10^12^ v.g./mL) were made by BrainVTA (Wuhan, China). Experiments were performed at least 4 weeks after virus injection. The mice that were injected with the above virus received a saline intraperitoneal injection (i.p.) to test the baseline WMC of the mice. Subsequently, to manipulate the neurons, CNO (1 mg/kg; A3317, APExBIO, USA) was administered to the mice via i.p. for viral infection.

### Slice electrophysiology

After 4 weeks of viral incubation, coronal sections, including PrL, were cut at a thickness of 300 μm in ice-cold cutting solution (185 mM sucrose, 2.5 mM KCl, 1.25 mM NaH_2_PO_4_·2H_2_O, 25 mM NaHCO_3_, 25 mM D-glucose, 0.5 mM CaCl_2_·2H_2_O, 10 mM MgSO_4_, oxygenated with 95% O2/5% CO_2_) using a vibrating microtome (VT 1200S, Leica, Germany). Next, slices were incubated in aerated (95% O2/5% CO_2_) artificial cerebrospinal fluid (ACSF: 125 mM NaCl, 2.5 mM KCl, 1.25 mM NaH_2_PO_4_·2H_2_O, 25 mM NaHCO_3_, 10 mM D-glucose, 2 mM CaCl_2_·2H_2_O, 1.5 mM MgSO_4_, pH 7.4), recovered at 32 °C for 30 min, and subsequently incubated at RT for 30 min. The pipettes were connected to the headstage of the Heka EPC 10 amplifier (Heka Elektronik, USA). Cells expressing mCherry in the PrL were identified on a microscope equipped with a differential interference contrast optical system (BX51WI, Olympus, Japan). Current-clamp recordings were applied to measure evoked action potentials in CNO activation and inhibition experiments. After applying current in 20 pA steps (from -60 to 300 pA), the neurons recovered for 5 min. Then, the slices were perfused with ACSF containing 5 μM CNO. The same procedure was performed 10 min after CNO perfusion.

### Statistical analysis

All data are presented as the mean ± standard error of the mean (SEM), and statistical tests were performed using GraphPad Prism version 9.0 software. Two-way analysis of variance (ANOVA) was used to analyze the effects of memory capacity and mouse genotype (wild-type or 5XFAD), the effects of current and CNO intervention, or the effects of memory capacity and virus intervention. Two-way ANOVA was also performed to analyze the effects of task and mouse genotype, and post hoc Tukey’s tests were applied to analyze the differences between groups. Linear regression was utilized to analyze the correlation between memory capacity and the number of FOS^+^ neurons, as well as the correlation between the number of FOS^+^ neurons and the number of FOS^+^ neurons co-localized with CaMKII^+^ neurons. We used Student’s *t* tests to analyze the other data. The statistical significance threshold for all tests was set at *p* < 0.05.

### Supplementary Information

**Additional file 1.** Individual values for figures. Each sheet in the Excel file is named by the figure.

## Data Availability

All data generated or analyzed during this study are included in this published article and its supplementary information files. The individual data values are provided in Additional file 1.

## Abbreviations

AD: Alzheimer's disease
WM: Working memory
WMC: Working memory capacity
PFC: Prefrontal cortex
mPFC: Medial prefrontal cortex
OWMC: Olfactory working memory capacity
PrL: Prelimbic cortex
IL: Infralimbic cortex
TG: 5XFAD
WT: Wild-type
NMSS: Nonmatching to a single odor sample
NMMS: Nonmatching to multiple odor samples
FOS^+^: FOS-positive
CaMKII^+^: Calmodulin-dependent protein kinase II-positive
GAD^+^: Glutamate decarboxylase-positive
CNO: Clozapine-N-oxide
E/I: Excitation and inhibition
PFA: Paraformaldehyde
BSA: Bovine serum albumin
RT: Room temperature
